# Efficacy of pazopanib monotherapy in patients who had been heavily pretreated for metastatic soft tissue sarcoma: a retrospective case series

**DOI:** 10.1186/s12885-015-1160-x

**Published:** 2015-03-19

**Authors:** Kwai Han Yoo, Hyo Song Kim, Su Jin Lee, Se Hoon Park, Sung Joo Kim, Soo Hee Kim, Yoon La Choi, Kyoo-Ho Shin, Yong Jin Cho, Jeeyun Lee, Sun Young Rha

**Affiliations:** Division of Hematology and Oncology, Department of Medicine, Samsung Medical Center, Sungkyunkwan University School of Medicine, 81 Irwon-ro, Gangnam-gu, Seoul, 135-710 Republic of Korea; Division of Medical Oncology, Department of Internal Medicine, Yonsei Cancer Center, Yonsei University College of Medicine, 50 Yonsei-ro, Seodaemun-Gu, Seoul 120-752 Republic of Korea; Department of Surgery, Samsung Medical Center, Sungkyunkwan University School of Medicine, Seoul, Republic of Korea; Department of Pathology, Yonsei Cancer Center, Yonsei University College of Medicine, Seoul, Republic of Korea; Department of Pathology, Samsung Medical Center, Sungkyunkwan University School of Medicine, Seoul, Republic of Korea; Department of Orthopedic Surgery, Yonsei Cancer Center, Yonsei University College of Medicine, Seoul, Republic of Korea

**Keywords:** Histological type, Pazopanib, Soft tissue sarcoma

## Abstract

**Background:**

We retrospectively reviewed outcomes of treatment with pazopanib, an oral multi-tyrosine kinase angiogenesis inhibitor, in patients with advanced soft tissue sarcoma, a rare and heterogeneous tumor group with limited treatment options.

**Methods:**

Between 2009 and 2013, 43 patients with metastatic soft tissue sarcoma received pazopanib as salvage chemotherapy after one or more cytotoxic regimens. Response rate, progression-free survival, and overall survival were analyzed according to histological subtype, Eastern Cooperative Oncology Group performance status, and metastatic site.

**Results:**

Common histological subtypes included leiomyosarcoma (n = 9), angiosarcoma (n = 6), malignant fibrous histiocytoma/undifferentiated pleomorphic sarcoma (MFH/UPS, n = 5), malignant peripheral nerve sheath tumor (MPNST, n = 5), and synovial sarcoma (n = 4). Nineteen patients (44.2%) received more than two chemotherapy regimens before pazopanib. At the time of analysis, 208 treatment cycles of pazopanib had been administered (median, 4.8 cycles per patient), and no treatment-related mortality occurred. The disease control rate was 61.0% (95% confidence interval [CI], 46.1–75.9%), and the overall response rate was 17.1% (partial response, n = 7; complete response, n = 0). Partial response was achieved in two patients with synovial sarcoma, two with MFH/UPS, one with MPNST, one with leiomyosarcoma, and one with angiosarcoma. The median lengths of progression-free survival and overall survival were 5.0 months (95% CI, 3.6–6.4 months) and 8.2 months (95% CI, 5.8–10.6 months), respectively. Progression-free survival was shorter in the patients with liposarcoma and rhabdomyosarcoma (1.3 and 0.9 months, respectively) than in those with leiomyosarcoma, MPNST, MFH/UPS, and synovial sarcoma (5.6, 6.5, 7.1, and 7.7 months, respectively).

**Conclusions:**

Pazopanib demonstrated acceptable antitumor activity in the Asian patients who had been heavily pretreated for sarcoma, with seemingly more favorable results in the patients with leiomyosarcoma, MPNST, MFH/UPS, and synovial sarcoma than in those with liposarcoma and rhabdomyosarcoma.

**Electronic supplementary material:**

The online version of this article (doi:10.1186/s12885-015-1160-x) contains supplementary material, which is available to authorized users.

## Background

Soft tissue sarcomas (STS) are a rare and heterogeneous group of tumors that originate from mesenchymal cells and account for 1% of all adult malignancies [1,2]. The primary treatments of STS are surgery and radiotherapy. However, approximately 40% of patients experience tumor recurrence, and survival rate is poor, with a median overall survival (OS) of less than 12 months [3]. STS is comprised of more than 50 different histological subtypes [4] that differ greatly in terms of treatment response and prognosis [2]. Despite this heterogeneity, patients with advanced non-gastrointestinal stromal tumor STS are generally treated in the same manner. However, chemosensitivity varies according to tumor subtype, and survival is also influenced by tumor grade, patient age, and performance status [5]. For instance, angiosarcoma is responsive to paclitaxel [6-9], and uterine leiomyosarcoma generally shows a good response to high-dose gemcitabine combined with docetaxel [10].Anthracyclines and ifosfamide, either alone or in combination, are the gold standard treatments of advanced STS [3,11]. However, after failure of conventional first-line cytotoxic chemotherapy, available treatment options are severely limited because of a high risk-to-benefit ratio in terms of patient tolerability and survival. Hence, the availability of less toxic agents that can improve chances of progression-free survival (PFS) or OS is crucial. In light of this, a recent pivotal phase III trial in refractory sarcoma patients (the PALETTE trial) demonstrated a significantly prolonged median PFS associated with pazopanib treatment.

Pazopanib is a synthetic indazolylpyrimidine that functions as a multitargeted tyrosine kinase inhibitor (TKI) with a high affinity for vascular endothelial growth factor receptors and a low affinity for platelet-derived growth factor receptors, fibroblast growth factor receptors, and c-Kit [12]. Based on the results of the PALETTE trial, pazopanib is currently recommended as the gold standard treatment for patients with metastatic non-adipocytic STS after failure of standard chemotherapy. However, only strictly selected patients with specific histological subtypes such as non-adiopocytic sarcoma were included in the clinical trial, and clinical use of pazopanib in unselected patients has not yet been fully documented. Furthermore, most patients enrolled in the PALETTE study were of Caucasian ancestry and only 57 of the 247 patients in the pazopanib group (23.2%) were from Asian ancestry (by personal communication with GSK). A recent phase II pazopanib trial demonstrated that Asian patients with nasopharyngeal carcinoma tolerated the treatment well, with a similar toxicity profiles [13]. However, the efficacy and tolerability of pazopanib in Asian patients with sarcoma are scarcely reported. Hence, the efficacy and tolerability of pazopanib in Asian patients should be evaluated. Therefore, we retrospectively investigated the anti-tumor efficacy of pazopanib in heavily pretreated STS patients from two major Korean cancer centers and conducted a subgroup analysis according to histological subtype.

## Methods

### Patients and treatment

We retrospectively reviewed the medical records of patients with advanced STS who were treated with pazopanib between May 2009 and November 2013 at Samsung Medical Center and Yonsei University College of Medicine, South Korea. The inclusion criteria were as follows: (1) pathologically confirmed STS and (2) availability of complete clinical information, including patient demographic characteristics, primary tumor site, tumor stage, and treatment record. The following clinicopathological variables were collected: age, sex, histological type, extent of metastasis, Eastern Cooperative Oncology Group (ECOG) performance status, and treatment history. The study was reviewed and approved by the Institutional Review Board of Samsung Medical Center and Yonsei University College of Medicine.

### Treatment

Pazopanib was administered orally at a dose of 800 mg once daily. The dose was reduced to 600, 400, or 200 mg for the management of adverse events at the discretion of the physician. Treatment was repeated every 4 weeks and continued until disease progression, unacceptable toxicity, or patient refusal. Evaluation of tumor response was performed every 2 months based on computed tomographic or magnetic resonance imaging findings. Responses were assessed according to the Response Evaluation Criteria in Solid Tumors, version 1.1 [14].

### Statistical analysis

Standard descriptive and analytical methods were used to describe the patient population and their baseline characteristics. OS was defined as the time from the initiation of pazopanib treatment to the date of death or last follow-up. PFS was defined as the time from initiation of the pazopanib treatment to the date of documented disease progression or death from any cause. Kaplan-Meier estimates were used to analyze time-to-event variables, and 95% confidence intervals (CIs) were computed for time-to-event medians. Survival comparisons were performed using univariate log-rank tests. The Cox proportional hazards model was used for multivariate analyses. Two-tailed *P* < 0.05 was considered statistically significant. Statistical analysis was performed using the SPSS version 18 software (SPSS Inc., Chicago, IL, USA).

## Results

### Patient characteristics

Between May 2009 and November 2013, 43 patients with relapsed or refractory STS were treated with pazopanib as at least a second-line chemotherapy. The patients’ baseline characteristics at the start of the pazopanib treatment are summarized in Table 1. The median age of the patients was 54 years (range, 19–74 years), and 60.5% of the patients were male. Most patients (86.0%) had a good ECOG performance status (i.e., 0 or 1). The distribution of histological subtypes was as follows: leiomyosarcoma (n = 9), angiosarcoma (n = 6), malignant fibrous histiocytoma/undifferentiated pleomorphic sarcoma (MFH/UPS, n = 5), malignant peripheral nerve sheath tumor (MPNST, n = 5), and synovial sarcoma (n = 4). FNCLCC (French Fédération Nationale des Centres de Lutte Contre le Cancer) grades were available for 22 patients (51.2%), of whom 13 (59.1%) had grade 3 sarcoma. The primary tumor site was the trunk or retroperitoneum in 34 patients (79.1%) and the extremities in 9 patients (20.9%). The most common metastatic sites were the lung (n = 32, 74.4%), bone (n = 17, 39.5%), and liver (n = 12, 27.9%).

**Table 1:** **Patient demographics and clinical characteristics**

		**n**	**%**
**Total**		**43**	**100**
**Age**	Median	54	---
	Range	19-74	---
**Gender**	Male	26	60.5
	Female	17	39.5
**ECOG PS**	0	7	16.3
	1	30	69.8
	2	6	14.0
**Histology**	Leiomyosarcoma	9	20.9
	Angiosarcoma	6	14.0
	MFH/UPS	5	11.6
	MPNST	5	11.6
	Fibroblastic tumor	4	9.3
	Synovial sarcoma	4	9.3
	Rhabdomyosarcoma	3	7.0
	Liposarcoma	2	4.7
	Tumor of uncertain differentiation	3	7.0
	Desmoplastic small cell tumor	1	2.3
	PEComa	1	2.3
**FNCLCC grade**	1	3	7.0
	2	6	14.0
	3	13	30.2
	unknown	21	48.8
**Primary site**	Trunk or retroperitoneum	34	79.1
	Extremity	9	20.9
**Organ involvement**	Lung	32	74.4
	Liver	12	27.9
	Bone	17	39.5
**Number of prior chemotherapy regimens chemotherapies**	1	9	20.9
	2	15	34.9
	≥3	19	44.2
**Agents used in prior regimen(s)**	Doxorubicin	30	69.8
	Ifosfamide	39	90.7
	Taxane	32	74.4
	Gemcitabine	28	65.1
	Dacarbazine	6	14.0
**Cycles of pazopanib treatment**	Median	4.8	---
	Range	1-17	---

ECOG = Eastern Cooperative Oncology Group, PS = performance status, MFH/UPS = malignant fibrous histiocytoma/undifferentiated pleomorphic sarcoma, MPNST = malignant peripheral nerve sheath tumor, PEComa = perivascular epithelioid cell tumor, FNCLCC = French Fédération Nationale des Centres de Lutte Contre le Cancer.

All of the patients had previously received at least one cytotoxic chemotherapy regimen before pazopanib treatment, and most of the patients had been heavily pretreated with at least two previous cytotoxic regimens. Fifteen patients (34.9%) had received two prior regimens, and 19 patients (44.2%) had received three or more prior regimens. In the study cohort, the following patients had received prior regimens: 69.8% (n = 30), doxorubicin-based chemotherapy; 90.7% (n = 39), ifosfamide-based chemotherapy; 74.4% (n = 32), taxane-based chemotherapy; and 65.1% (n = 28), gemcitabine-based chemotherapy.

Pazopanib therapy was administered in 208 treatment cycles, with a median of 4.8 cycles per patient (range, 1–17 cycles). At the time of analysis, 6 patients were still receiving pazopanib treatment, and the remaining 37 patients had stopped treatment because of progression (n = 30), patient refusal (n = 1), toxicity (n = 1), or other reasons (n = 5).

The most common non-hematological toxicities were diarrhea (grade 3 or 4, n = 2), hand-foot syndrome (grade ≥ 2, n = 6), and anorexia (grade 3 or 4, n = 2). The grade 3 or 4 hematological toxicities were neutropenia (n = 1) and thrombocytopenia (n = 1). No treatment-related mortality or neutropenic fever was associated with pazopanib treatment. Most non-hematological toxicities were reversible with appropriate medical management (e.g., loperamide for diarrhea) and dose reduction. The pazopanib dose was reduced in 48.8% of the patients (n = 21).

### Tumor responses

**Figure 1:**
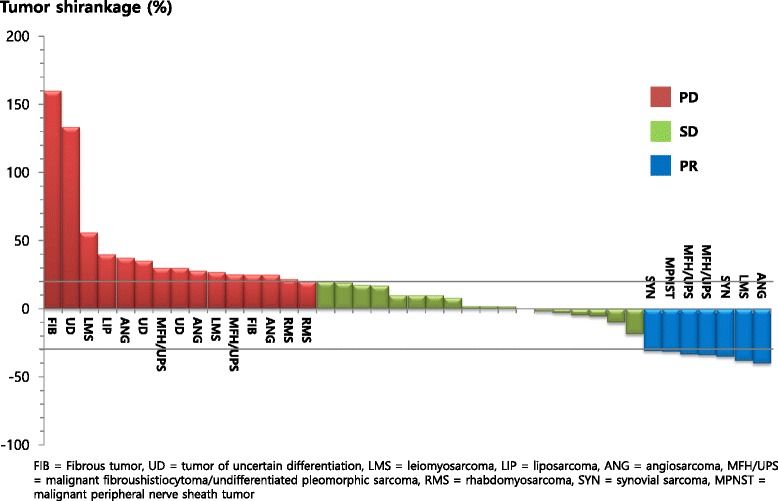
**Maximum response to pazopanib treatment.**

Of the 43 patients, 41 were evaluable for tumor response. Figure 1 shows the maximum response after pazopanib treatment. None of the patients achieved a complete response, but seven patients achieved partial responses, yielding a 17.1% (95% CI, 5.6–28.6%) overall response rate. Responses were observed in the patients with synovial sarcoma (n = 2, 50%), MFH/UPS (n = 2, 40%), MPNST (n = 1, 20%), leiomyosarcoma (n = 1, 11%), and angiosarcoma (n = 1, 17%). Of the seven patients with partial responses, three maintained treatment response for more than 10 cycles at the time of this writing. Eighteen patients (43.9%) achieved stable disease, at a disease control rate of 61.0% (95% CI, 46.1–75.9%). Thus, objective tumor response or stable disease for more than 12 weeks was attained in 51.2% of the patients (n = 22; 95% CI, 43.6–58.8%), and the median response duration was 251 days (range, 30–469 days).

### Survival outcomes

At a median follow-up of 12.0 months (95% CI, 5.7–18.3 months), the median PFS was 5.0 months (95% CI, 3.6–6.4 months), and the median OS was 8.2 months (95% CI, 5.8–10.6 months). We analyzed PFS and OS in patients with different histological subtypes (Table 2). Patients with liposarcoma or rhabdomyosarcoma had significantly poorer survival when compared with patients with non-liposarcoma or non-RMS cases (*P* < .001, Additional file 1: Figure S1A). Accordingly, the patients with liposarcoma (n = 2) had poorer survival after pazopanib treatment than those with other types of sarcoma (Additional file 1: Figure S2A). Of note, the patients with RMS progressed to pazopanib rapidly, but were all alive at the time of this writing. Age (>50 years *vs* ≤50 years, *P* = 0.079), number of prior chemotherapy regimens (≥2 *vs* 0–1, *P* = 0.162), primary site (truck/retroperitoneum *vs* extremity, *P* > 0.05), and existence of liver and lung metastases (presence *vs* absence, *P* = 0.756) were not significantly correlated with PFS at univariate level. However, performance status (ECOG 2 *vs.* 0 or 1, *P* = 0.034) and prior response to doxorubicin (stale response or progression *vs* partial response, *P* = 0.05) at the time of pazopanib treatment were significant adverse factors of pazopanib treatment in terms of PFS.

**Table 2:** **Survival of patients with various histologic subtypes**

**Histology**	**n**	**%**	**PFS [months, (95% CI)]**	**OS [months, (95% CI)]**
Leiomyosarcoma	9	20.9	5.6 (2.7-8.5)	9.9 (5.3-14.4)
Angiosarcoma	6	14.0	3.2 (2.1-4.2)	8.0 (3.7-12.4)
MFH/UPS	5	11.6	7.1 (0.3-13.8)	7.5 (1.1-13.9)
MPNST	5	11.6	6.5 (0.7-12.3)	8.9 (3.5-14.3)
Fibroblastic tumor	4	9.3	5.7 (0.7-10.8)	6.4 (1.6-11.2)
Synovial sarcoma	4	9.3	7.7 (3.5-11.9)	9.4 (4.4-14.5)
Rhabdomyosarcoma	3	7.0	0.9 (0.5-1.3)	2.5 (0.7-4.4)
Liposarcoma	2	4.7	1.3 (0.7-1.9)	1.5 (1.1-1.8)
Tumor of uncertain differentiation	3	7.0	1.4 (0.5-2.3)	4.1 (−0.4-8.5)
Desmoplastic small cell tumor	1	2.3	10.3 (N/A)	43.4 (N/A)
PEComa	1	2.3	3.8 (N/A)	4.1 (N/A)
Total	43	100	5.0 (3.6-6.4)	8.2 (5.8-10.6)

PFS = progression-free survival, CI = confidence interval, OS = overall survival, MFH/UPS = malignant fibrous histiocytoma/undifferentiated pleomorphic sarcoma, MPNST = malignant peripheral nerve sheath tumor, PEComa = perivascular epithelioid cell tumor; N/A, not available.

## Discussion

We demonstrated that pazopanib is a feasible treatment option with acceptable antitumor activity in patients who had been heavily pretreated for metastatic STS. Considering that a few patients in the previous clinical trials were of Asian ethnicity [15-17], our study also shows that pazopanib is well tolerated in Asian STS patients.

Beyond standard systemic treatment with doxorubicin and ifosfamide, newer agents such as gemcitabine, trabectedin, and pazopanib have been assessed for subsequent treatment of STS. In a phase III trial, pazopanib treatment significantly improved PFS compared with placebo, with a median prolongation of 3 months [16]. These results led to the approval of pazopanib for STS, making it the first anti-angiogenic drug approved for STS. In terms of efficacy, our study shows similar outcomes (PFS, 5.0 months and OS, 8.2 months) as those of the PALETTE trial. However, our observation of shorter OS compared with that in the PALETTE trial might be because most of our patients had been heavily pretreated with at least two prior chemotherapy regimens. Regarding toxicity profile, despite the retrospective nature of our study, we used the same dosage and dose modification protocol as those used in the PALETTE trial. Similar frequency of dose reduction (48% in our cohort *vs* 39% in the PALLETE trial) and treatment duration (median, 19 weeks *vs* 16.4 weeks) were observed.Given the diverse histological subtypes of STS with different biological and clinical behaviors, an exploratory subgroup analysis according to histological subtype was performed in previous trials [15,16]. In the EORTC 63043 study, 142 patients were recruited into four different strata as follows: adipocytic STS, leiomyosarcoma, synovial sarcoma, and other STS types. Among the strata, the adipocytic sarcoma stratum was closed due to poor PFS rate at 12 weeks. Based on this phase II trial, adipocytic STS was excluded from the PALETTE trial. In a recent subgroup analysis of long-term responders to pazopanib [17], patients with leiomyosarcomas and synovial sarcomas, vascular tumors, alveolar soft part sarcoma, solitary fibrous tumors, and desmoplastic small round cell tumors were the main long-term responders and survivors. In line with these results, we also observed better anti-tumor activity of pazopanib in patients with non-adipocytic sarcoma such as leiomyosarcoma, MPNST, MFH/UPS, or synovial sarcoma but less activity in patients with adipocytic sarcoma or rhabdomyosarcoma.

With regard to treatment response to angiogenesis inhibitors such as pazopanib among patients with adipocytic sarcoma, several trials have reported varying results from subgroup analyses [16,18,19]. Similar to the PALETTE trial, a phase II trial demonstrated that patients with liposarcoma were poor responders to sorafenib [18]. By contrast, patients with liposarcoma showed a promising treatment response to sunitinib in a single-institution study, with 3.9 months of PFS and 18.6 months of OS[19]. Currently, the underlying biological mechanism of poor response to pazopanib in adiopocytic sarcoma has not been defined, but it may depend on the heterogeneity of adipocytic sarcoma subtypes (i.e., dedifferentiated, myxoid, round cell, and pleomorphic). In our previous work, we showed a distinct prognosis and the clinical features of dedifferentiated liposarcoma among other adipocytic sarcomas [20]. However, as all but two liposarcoma patients included in this analysis had myxoid liposarcoma, we cannot draw definitive conclusions regarding treatment response to pazopanib according to different subtypes.

In terms of pediatric sarcomas, clinical activity with pazopanib was demonstrated for desmoplastic small round cell tumors and alveolar rhabdomyosarcoma in a phase I study [21]. In our case series, poor responders were patients with embryonal and spindle cell rhabdomyosarcoma who had failed to respond to four previous cytotoxic regimens. Because these patients already had refractory sarcoma at the time of pazopanib therapy, it is difficult to define the anti-tumor activity of pazopanib in this subset. However, these patients failed to respond to pazopanib within 2 months of therapy initiation, which suggests that pazopanib is not an optimal treatment option for this subtype. Nevertheless, because of our small sample size, further observations are required regarding the effect of pazopanib in adult-onset pediatric type sarcoma.

Some limitations of this study include its retrospective nature and small sample size, especially for the histological subgroup analyses. However, to date, no study reports the efficacy of pazopanib specifically in an Asian population. Furthermore, unlike well-controlled clinical trials [15,16], our patient cohort was mostly composed of individuals with poor performance status who had been heavily pretreated.

## Conclusion

In summary, we demonstrated that pazopanib is a feasible option for the patients who had been heavily pretreated for metastatic sarcoma, with a small subset of patients achieving long-term responses. To gain insight into the subgroup of patients who could benefit most from pazopanib, an expansion of the patient pool through a large multicenter cohort study is needed.

## Additional file

Additional file 1: Figure S1. Survival according to histological subtype. A) Progression-free survival (PFS) and B) overall survival (OS).

## Abbreviations

STS: Soft tissue sarcoma
OS: Overall survival
PFS: Progression-free survival
TKI: Tyrosine kinase inhibitor
ECOG: Eastern Cooperative Oncology Group
CI: Confidence interval
MFH/UPS: Malignant fibrous histiocytoma/undifferentiated pleomorphic sarcoma
MPNST: Malignant peripheral nerve sheath tumor
FNCLCC: French Fédération Nationale des Centres de Lutte Contre le Cancer
