# Supplementary figures and images for: Efficacy of pazopanib monotherapy in patients who had been heavily pretreated for metastatic soft tissue sarcoma: a retrospective case series
Source: BMC Cancer. 2015 Mar 19;15:154. doi: 10.1186/s12885-015-1160-x (PMC4438639; doi:10.1186/s12885-015-1160-x)

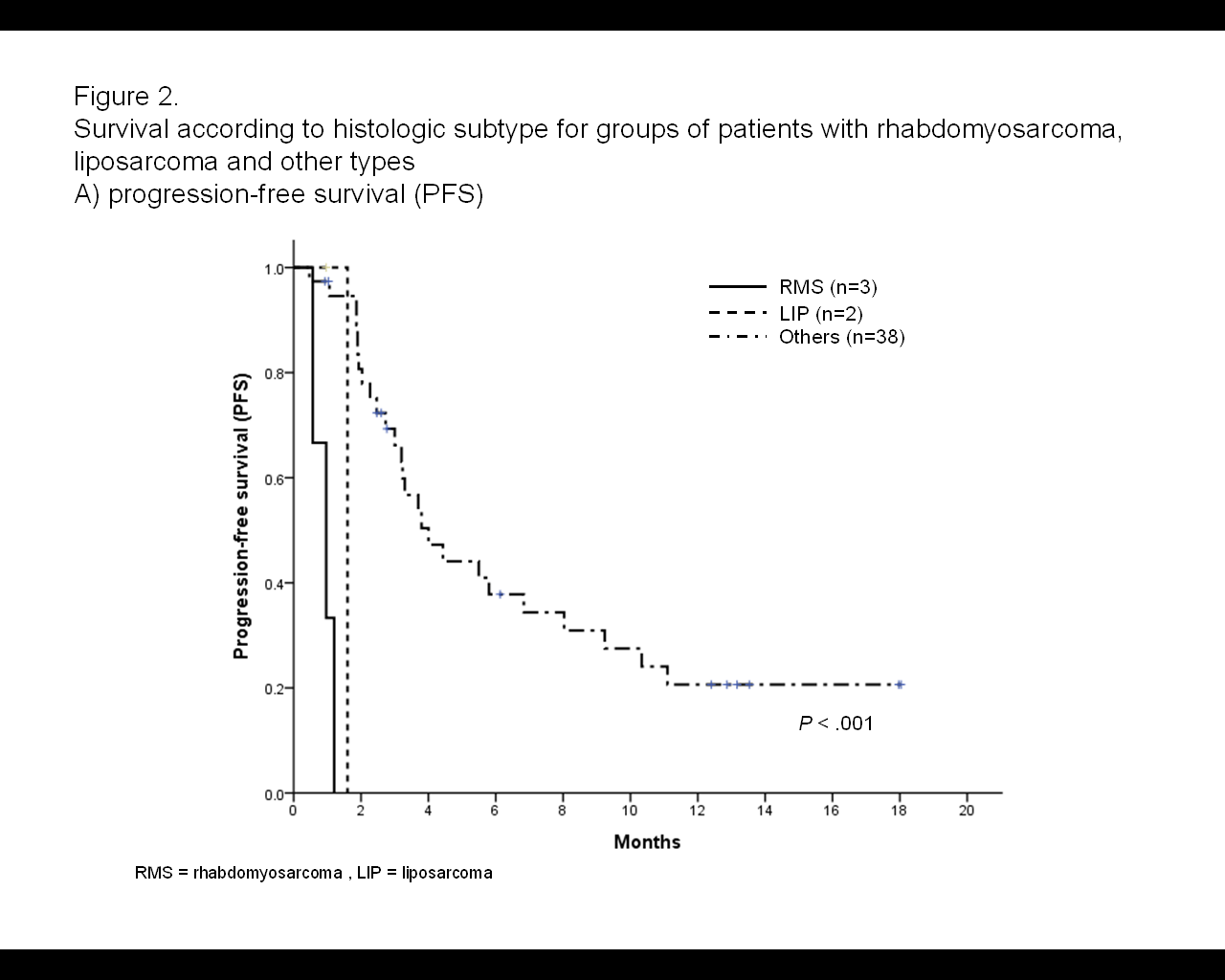


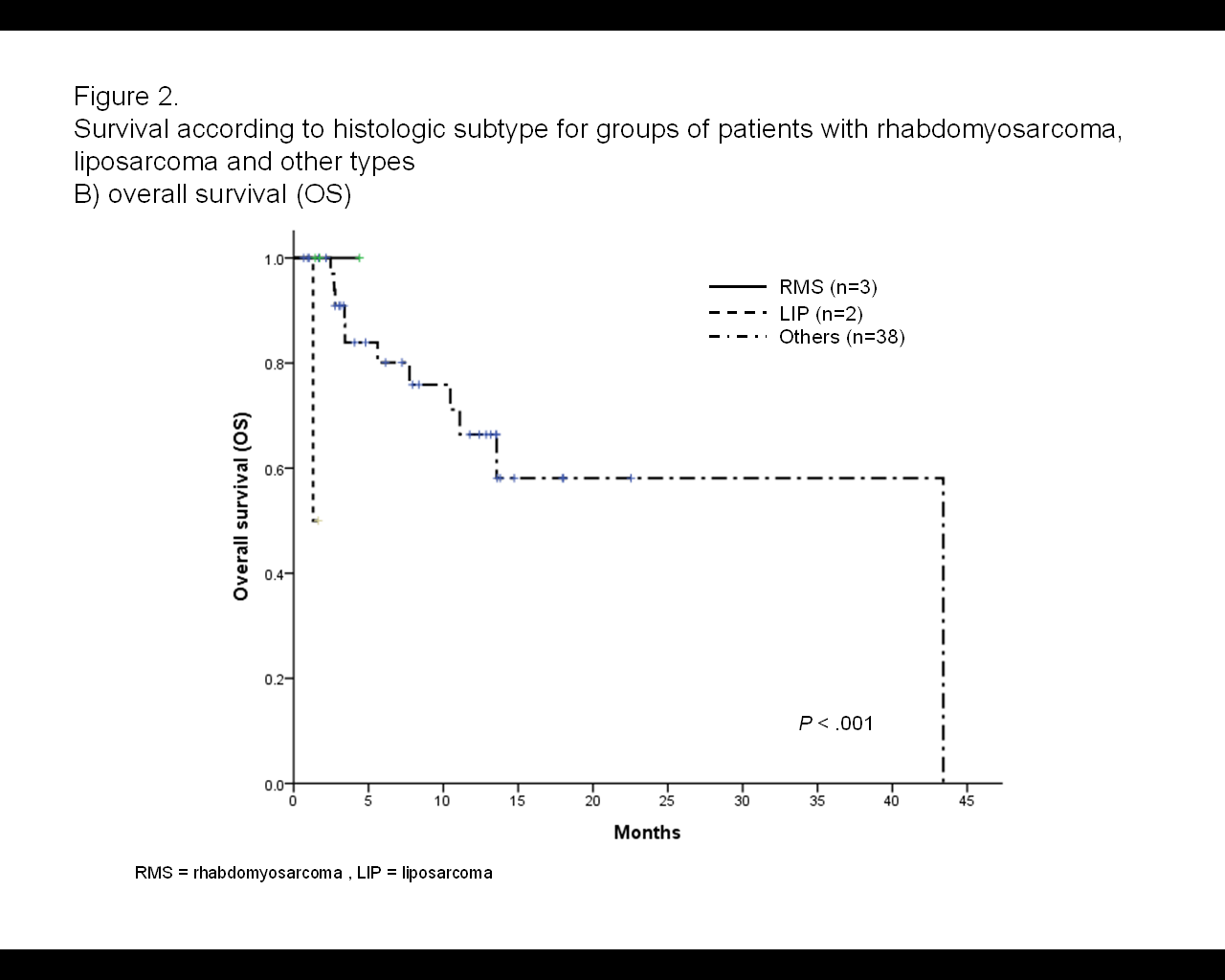

Supplement: Additional file 1: Figure S1. — Survival according to histological subtype. A) Progression-free survival (PFS) and B) overall survival (OS). [file 12885_2015_1160_MOESM1_ESM.doc]
